# Infertility: Practical Clinical Issues for Routine Investigation of the Male Partner

**DOI:** 10.3390/jcm9061644

**Published:** 2020-05-30

**Authors:** Alberto Ferlin, Carlo Foresta

**Affiliations:** 1 Department of Clinical and Experimental Sciences, Unit of Endocrinology and Metabolism, University of Brescia and ASST Spedali Civili Brescia, 25123 Brescia, Italy; 2 Department of Medicine, Unit of Andrology and Reproductive Medicine, University of Padova, 35121 Padova, Italy; carlo.foresta@unipd.it

**Keywords:** asthenozoospermia, azoospermia, male infertility, oligozoospermia, semen analysis, sperm, testes

## Abstract

About one-fifth of couples has fertility problems in Western countries. Male factors are present in about half of them, either alone or in combination with female causes. Therefore, both partners should be evaluated simultaneously. The fertility status and/or specific conditions of each partner influence the clinical and treatment approach. This article summarizes in a practical way when, how, and why the male partner of an infertile couple should be investigated. The available evidence and international guidelines were used, interpreting, discussing, and expanding them from personal decades-long experience in this field. The aim is to delineate the most appropriate clinical approach for the male partner of infertile couples, considering traditional and emerging technologies and laboratory analyses in the context of their clinical significance. Components of the initial evaluation in men without known risk factors for infertility should include at minimum medical history, physical examination, and semen analysis. Semen microbiological examination, endocrine assessment, scrotal ultrasound, and transrectal ultrasound are suggested in most men and are mandatory when specific risk factors for male infertility are known to be present or when the initial screening demonstrated abnormalities. Full examination, including genetic tests, testicular histology, or additional tests on sperm, is clinically oriented and/or suggested after the results of initial investigations.

## 1. Introduction

In Western countries, approximately 15–20% of couples are infertile as defined by the inability to conceive after one year of unprotected intercourse. Male factors are present in about half of infertile couples, either alone or in combination with female causes [1]. In recent years, technology and assisted reproduction erroneously prompted the concept that full medical investigation for infertile men is not necessary and, on the other hand, male infertility is often defined only based on semen analysis. Indeed, male infertility can be caused by a variety of aetiologies, and semen parameters merely represent the end point of different pathophysiologic mechanisms. Although guidelines for the optimal diagnostic evaluation of male infertility exist [1,2,3,4,5,6,7], there is no general consensus on the best clinical workup for the male partner of an infertile couple based on evaluation of the fertility potential and risk factors of both partners and taking into account diagnostic tests developed more recently for analyzing sperm function and causes of spermatogenic impairment.

The objective of this article is to summarize in a practical way when, how, and why the male partner of an infertile couple should be investigated. The focus is to delineate the most appropriate clinical and diagnostic approach for the male partner of infertile couples, considering traditional and emerging technologies and laboratory analyses in the context of their clinical significance. To this aim, we referred to available evidence and international guidelines, and interpreted, discussed, and expanded them from our personal decades-long experience in this field.

## 2. When the Male Partner Should be Investigated and Goals of Evaluation

Infertility is not a matter of the woman or the man. Indeed, “male infertility” should be better defined as “male factor infertility” when in an infertile couple a specific cause or risk factor explaining the infertility status of the couple is identified in the male partner. Similarly, too often a definition of “idiopathic infertility” is used, but it is quite evident that the term idiopathic should be reserved to cases in which there is no identifiable cause after a detailed and meticulous diagnostic process.

A typical malpractice when approaching an infertile couple is to focus the attention just to one of the partners and/or limit the diagnostic process of the male partner to semen analysis. Both partners of couples experiencing problems in conceiving should be evaluated simultaneously, and the fertility status of each partner and/or specific conditions present in a member of the couple influence the clinical and treatment approach. For example, poor fertility in a male partner could be compensated by excellent fertility status in the female partner and, alternatively, apparent good fertility in the male partner could not be sufficient to overcome the poor fertility of the female partner. Laboratory tests should be interpreted in the context of that particular couple and not as independent predictor of (in) fertility. As example, mild oligozoospermia could be compatible with fertility when the female is at her top fertility capability (e.g., 25 years old) or might be a cofactor of infertility when the fertility potential of the female is drastically reduced (e.g., 40 years old).

Gynaecologists and andrologists should proceed jointly in deciding the clinical approach of the infertile couple in order to have both partners screened via a standard protocol. From this initial screening, more specialized investigations can be planned on the male and/or female partner. Limiting the investigation to one partner or having minimal investigation in one partner when one component of the couple has already a diagnosis of infertility could delay diagnosis, proper treatment, and time to pregnancy. Ideally, a personalized investigation approach based on individual components of the couple and the global assessment of the fertility potential of the couple is needed.

Importantly, infertility is a retrospective diagnosis, as it is defined by the World Health Organization (WHO) as the failure to achieve a clinical pregnancy after 12 months or more of regular unprotected sexual intercourse [2,3,4,5,6,7,8]. Therefore, one basic question is related to the timing for the initial investigation, because it is evident that we cannot wait one year in all cases to start the diagnostic process when the couple is asking us whether an evaluation is needed before 12 months have passed. Sometimes, we can reassure the couple and postpone diagnostic evaluation, but we can do this only when the couple is young and no risk factors for infertility from both sides are present. Earlier evaluation is warranted when specific risk factors for male infertility are known to be present (Table 1) or low fertility in the female partner is highly suspected (including advanced age). In addition, men who question their fertility status despite the absence of a current partner should have an evaluation [2,3,4,5,6,7,8].

The goal of investigation is to have a diagnosis based on etiologic and pathophysiologic mechanisms, which is required for appropriate treatment and has prognostic value for the fertility outcome of the couple.

**Figure 1:**
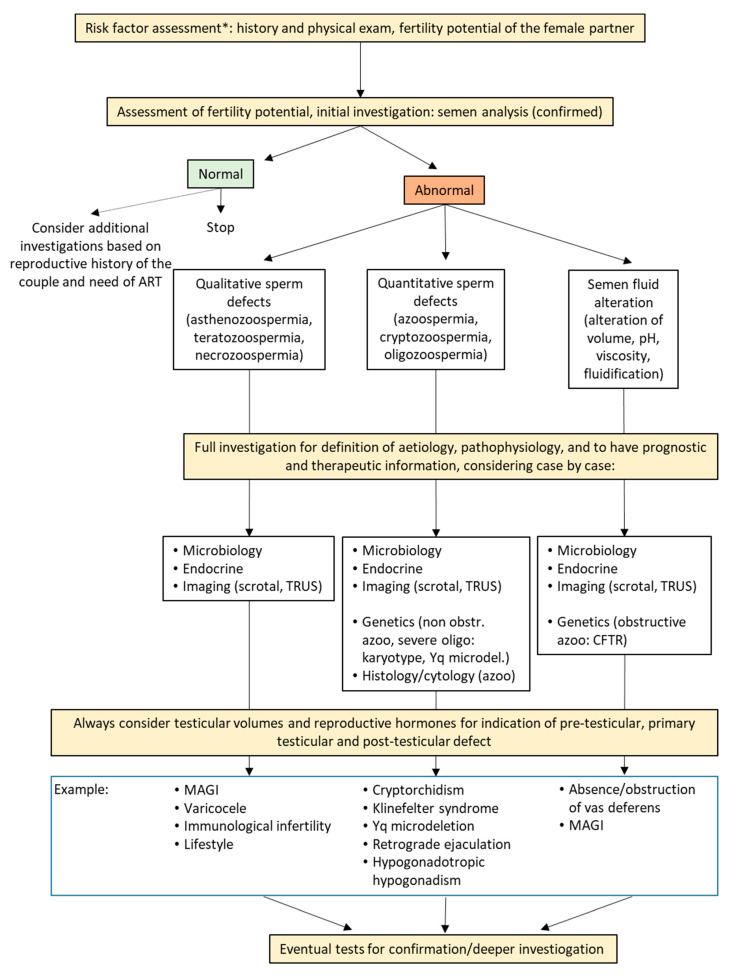
Schematic diagnostic flow chart for male factor infertility. ART: assisted reproduction technique; *CFTR*: cystic fibrosis transmembrane regulator; MAGI: male accessory gland inflammation; TRUS: transrectal ultrasound. * If risk factors are present, initial evaluation should already include full investigarion (microbiology, endocine, imaging, genetic), as appropriate.

Components of the initial evaluation in men without known risk factors for infertility should include at minimum medical history, physical examination, and semen analysis [1,2,3,4,5,6,7,8,9,10,11]. Semen microbiological examination, endocrine assessment, and imaging are useful investigations in most men and are mandatory when specific risk factors for infertility exist or the initial screening demonstrated abnormalities. Full examination including also genetic tests, testicular histology, or additional tests on sperm is clinically oriented and based on the results of previous investigations (Figure 1).

## 3. Initial Clinical Approach: History and Physical Examination

Accurate medical history and physical examination are mandatory in the initial approach to the male partner of an infertile couple. The known causes and risk factors listed in Table 1 should be questioned in detail with a general medical history and a specific andrological and reproductive history. Furthermore, sexual history should be evaluated to verify timing and frequency of sexual intercourses and possible sexual problems (libido, erection, ejaculation) (Table 2).

Physical examination should include genital examination and the assessment of secondary sex characteristics, other than some general features (Table 3). Of particular importance is testicular volume, which has a positive correlation with sperm count and guides on the origin of infertility (pre-testicular, testicular, post-testicular). Digital rectal examination could be useful especially in patients with suspected genital tract infections and inflammation or in the presence of specific semen abnormalities (high viscosity, low, or high pH, low or high semen volume, leukocytospermia).

## 4. Semen Analysis: The Mainstream for Further Decision-Making Investigations

No conclusions about the fertility potential of a man could be drawn without a semen analysis [1,2,3,4,5,6,7,8,9,10,11]. However, it should be clear that semen analysis is not necessary equivalent to the fertility potential of a man, and in particular of the specific couple you are investigating. Importantly, semen analysis gives information on the status of the entire seminal tract; therefore, it gives indications for further analyses other than fertility assessment. Importantly, semen analysis is not the final factor in a diagnosis of male factor infertility [12,13], and many aetiologies and different pathophysiological mechanisms might result in the same semen alteration. Semen analysis merely indicates the fertility potential and health status of the testes and seminal tract. Accordingly, no therapies should be initiated only based on semen analysis alone [14,15]. Furthermore, the information obtained from semen analysis should derive from a complete evaluation of all the parameters (macroscopic evaluation, microscopic evaluation of sperm, and non-spermatogenic cells) and in the context of all the other clinical information of that particular patient. A common malpractice is to look only at sperm concentration, motility, and morphology. To this regard, a practical aspect is to consider total sperm count per ejaculate, rather than sperm concentration per milliliter, because it better reflects testicular and seminal tract function [16]. Furthermore, total sperm count has been recently demonstrated to reflect the general health of a man [12].

Semen analysis should be performed following the WHO recommendations [16] by trained personnel in a specialized laboratory that follows strict internal and external quality control programs. Pre-analytical, analytical, and post-analytical factors could interfere with the reliability of the analysis and should be considered when interpreting the report. Natural variation in semen analysis occurs; therefore, no diagnosis could be made until two analyses have been performed. As spermatogenesis lasts more than 2 months, the interval between the two semen analyses should ideally be 2–3 months when acute illnesses or medical treatment interfering with spermatogenesis occurred in the last 2–3 months.

From a clinical point of view, it is important to note that WHO semen criteria are not “normal values” but rather “reference values” derived from a population-based study of less than 2000 fathers (time to pregnancy ≤12 months) and are expressed as percentiles (Table 4). Therefore, references values are just indications of the fertility status of a man; parameters in the 95% confidence interval do not guarantee fertility, and on the contrary, men who have semen parameters below the fifth percentile are not necessarily infertile [16]. For example, this also implies that no clinical indications or judgment on fertility should be present in the semen analysis report. As said above, the same semen parameters might have different clinical significance depending on the fertility status of the female partner.

Caution should be made also when interpreting the reference values. The WHO considers only the lower limit (fifth percentile) for semen parameters, but it is obvious that for some parameters, more is not always better (for example, high semen volume and high pH usually suggest prostate and/or seminal vesicles dysfunction).

Abnormalities in semen analysis could be grossly categorized in three main groups: quantitative sperm defects, qualitative sperm defects (Table 5), and semen fluid alterations (including abnormalities of semen volume, pH, viscosity, fluidification). A confirmed finding of one of these abnormalities suggests that additional tests are necessary to reach clinical conclusions (Figure 1).

Azoospermia should be defined only after centrifugation of the semen specimen and analysis of the entire pellet to distinguish it from cryptozoospermia [1,16]. Finally, we prefer to include analysis of sperm antibody as first line assessment, although guidelines do not agree as to whether this evaluation should be included as a second step [2,3,4,5]. In our experience, about 5% of infertile men show sperm auto-antibodies, which is in agreement with large series [3]. Analysis of biochemical parameters (zinc, fructose, neutral glucosidase) are optional procedures [16].

## 5. When and Why Second-Line Investigation is Indicated: The Role of Semen Microbiological, Endocrine, and Imaging Assessment

Semen microbiological examination is important to show infections of the urogenital tract, which could be present in testis, epididymis, prostate, and seminal vesicles. All these conditions go under the term of MAGI (male accessory gland infection) and can interfere with quantitative or qualitative alterations of spermatogenesis and sperm function [1,17]. Results of semen culture should be considered in the context of the other clinical information and above all on imaging studies (ultrasound of the testis and epididymis, transrectal ultrasound for prostate and seminal vesicles). Other than general semen culture for bacteria, Mycoplasma, and Chlamydia, recent evidence suggest also to consider infection for Human Papilloma Virus (HPV), especially in men with risk factors for this infection and/or those with asthenozoospermia, the presence of antisperm antibody and repeated abortion or failure of the assisted reproduction techniques (ART) procedure [18].

Endocrine assessment is essential for a correct diagnosis in most cases and above all when quantitative sperm defects exist. Furthermore, it is particularly indispensable for suggesting treatment approach (for example, high follicle stimulating hormone (FSH) clearly talks against FSH treatment) [14] and should also be considered in the whole clinical context of the patient (history, testicular volumes, associated co-morbidities, etc.).

Information on the hypothalamic–pituitary–testicular (HPT) axis is easily performed, as initial step, by follicle stimulating hormone (FSH), luteinizing hormone (LH), and total testosterone morning serum concentrations [1,2,3,4,5,6,7,8,9,10], which reflect the two inter-related functions of the testis: spermatogenesis and androgen production. The HPT axis could be affected at different levels by different causes, mainly at the hypothalamic–pituitary level (secondary hypogonadism) or at the testicular levels (primary hypogonadism and subclinical hypogonadism) [12]. Furthermore, combined analysis of both testicular axes (FSH: Sertoli cell, LH: Leydig cells) in association with total sperm count give a better indication of the male general health [12]. In cases of primary testiculopathy, no other endocrine assessment is generally required, whereas in cases of central, secondary hypogonadism, other pituitary hormones and endocrine axis should be investigated (e.g., prolactin, Thyroid Stimulating Hormone (TSH), and thyroid hormones, growth hormone), as well as pituitary MRI should be hypothesized depending on clinical data and aetiology. In specific cases (such as obesity, chronic liver disease, or total testosterone levels in the borderline range), sex-hormone binding globulin (SHBG) should be measured and free testosterone calculated [1]. A guide for interpreting basic reproductive hormone findings is reported in Table 6.

Scrotal ultrasonography allows having more precise information than physical examination. Testis volume is accurately determined, as well as morphology, ultrasound pattern, testis position, quantification of eventual hydrocele, the presence of microlithiasis, and vascularization [1,9,10,19]. Association with color Doppler allows detecting and quantifying varicocele. Furthermore, scrotal ultrasound is necessary to adequately assess epididymal and vas deferens morphology, dilation in cases of obstruction, absence, signs of inflammation/infection such as cysts, and it is of great aid when epididymal sperm aspiration is programmed for future assisted reproduction techniques (ART) [19]. Given also the strong association between infertility, cryptorchidism, testicular hypotrophy, and microlithiasis with testicular cancer, scrotal ultrasonography is a great opportunity to identify suspected testis masses and nodules [19,20].

Transrectal ultrasound (TRUS) is suggested in cases of suspected absence/obstruction of vas deferens, ejaculatory duct obstruction, and to have information on prostate and seminal vesicles in cases of suspected MAGI [17,19]. Therefore, it could give important information when both quantitative and qualitative sperm defects exist, as well as when semen fluid alterations and/or leukocytospermia or semen infection are present.

## 6. When and Why Third-Line Investigation is Indicated: The Role of Genetic Testing and Testicular Histology/Cytology

Thousands of genes are implicated in spermatogenesis, testicular development, and endocrine regulation of testicular function. Genetic causes of male infertility vary from chromosomal abnormalities to copy number variations (CNVs), to single-gene mutations [21]. Therefore, the genetic contribution to male factor infertility is considerable, and new genetic causes and genetic risk factors for male factor infertility have been identified in recent years, pushed by new technologies for genetic analysis. Therefore, it is presumed that other genetic and epigenetic tests will be introduced in clinical practice in the near future. Meanwhile, routine genetic tests suggested in the clinical practice are relatively few [21]. However, it is important that they should be performed in a targeted approach in selected cases to minimize unnecessary investigation [21,22,23]. In fact, the most important aspect is related to the correct identification of subjects to be tested and the right application of genetic tests based on clear clinical data. A correct application of available genetic tests allows receiving a better and defined diagnosis, has an important role in clinical decision (treatment, prognosis), and allows appropriate genetic counseling, especially in cases that should undergo ART to assess the risk of the couple to transmit its genetic characteristics.

Cytogenetic testing (karyotype) and Y chromosome microdeletion analysis are recommended in patients with azoospermia and severe oligozoospermia (<10 million sperm/ejaculate) due to primary testicular failure (generally, normal semen volume and pH, testicular hypotrophy and normal/high FSH) [1,22,23,24,25,26].

On the contrary, obstructive azoospermia with bilateral congenital absence of vas deferens (CBAVD) (generally, low semen volume and pH, normal testicular volume, normal reproductive hormones, no evidence of vas deferens by palpation, and scrotal and transrectal ultrasonography) should be tested for cystic fibrosis transmembrane conductance regulator (*CFTR*) gene mutations (including the 5Tallele) [1,21,22,23,24,27,28]. Mutations in *CFTR* might be associated also with unilateral absence of vas deferens (CUAVD). In this condition, semen analysis, testicular volumes, and hormonal levels are normal if the testis of the unaffected side is normally functioning. Therefore, suspect is derived by palpation of the vas deferens or, better, by scrotal and transrectal ultrasonography. Whenever the couple is planning a pregnancy by ART, the *CFTR* test should be performed in at least one of the partners because of the high prevalence of *CFTR* mutations in the general population [24,27].

Other genetic analyses that could be considered are related to specific clinical condition, other diagnostic tests, and availability of laboratories performing the tests. Mutation analysis of the androgen receptor (*AR*) gene is suggested in cases of non-obstructive azoospermia and severe oligozoospermia with evidence of androgen insensitivity (high/normal testosterone and high LH) [21,24]. Rare causes of male infertility (spermatocytic arrest, isolated idiopathic complete asthenozoospermia, globozoospermia, macrocephaly) could be tested for specific gene mutations (*TEX11*, dynein genes *DNAI1*, *DNAH5*, *DNAH11*, *DPY19L2*, *AURKC*, respectively) [21,29]. Mutation analysis of *INSL3*/*RXFP2* genes has been suggested in patients with a history of cryptorchidism [21,30,31], and mutations in the *NR5A1* gene are emerging as a significant cause of primary spermatogenic impairment associated or not with cryptorchidism [32]. New technologies will allow in a near future to test many genes through gene panels [33]. This is already suggested for the screening of tens of genes implicated in hypogonadotropic hypogonadism [1,21]. Finally, pharmacogenetic tests for FSH treatment (polymorphisms in *FSHB* and *FSHR* genes) are promising but not yet applicable routinely on clinical practice [34,35,36,37].

In cases of azoospermia, clear distinction between obstructive and non-obstructive forms is fundamental for further clinical and therapeutic approach. History, testicular volume, semen volume and pH, scrotal ultrasound, TRUS, and endocrine assessment in most cases allow having indication to this regard [11]. In particular, non-obstructive azoospermia is suggested from a combination of bilateral testicular hypotrophy, normal semen volume and pH, high FSH levels, reduced intratesticular vascularization, inhomogeneous echo-texture, and normal epididymes at scrotal colour Doppler ultrasound, normal results at TRUS. History also might suggest primary testicular damage, such as in cases on cryptorchidism, testicular trauma, orchitis, testicular torsion, chemotherapy, or known Klinefelter syndrome. On the contrary, obstructive azoospermia is suggested from a combination of normal testicular volumes, reduced semen volume and alterations in pH, normal reproductive hormone levels, normal testicular patter with dilated epididymes or absence/obstruction of vas deferens at scrotal colour Doppler ultrasound, abnormal results at TRUS (for example, ejaculatory duct obstruction, absence of seminal vesicles), and known CFTR gene mutation. However, the gold standard in distinguishing obstructive and non-obstructive forms is histopathology analysis of the testes [3,8]. Furthermore, in cases of non-obstructive azoospermia, different spermatogenic alterations might be present, with different prognostic value: Sertoli cell-only syndrome (complete absence of spermatogenesis), hypospermatogenesis (quantitative reduction of germ cells), and germ cell maturation arrest (at the spermatogonia, spermatocyte or spermatid level). These conditions cannot be clearly distinguished by testis volume and FSH levels. Of particular note, a good practice is to associate testicular biopsy with the cryopreservation of sperm, in order not to repeat testicular sperm retrieval at the time of Intracytoplasmic Sperm Injection (ICSI) [8].

Cryptozoospermia and severe oligozoospermia might also benefit from histopathology analysis, although in these cases, sperm cryopreservation might be done usually from semen. In addition to these cases, the specific spermatogenesis alteration (hypospermatogenesis, maturation disturbances, partial obstructive forms) cannot be predicted from other investigations, and therefore, this analysis allows for a more precise diagnosis and prognosis (for example, FSH treatment is better suggested when hypospermatogenesis without associated maturation arrest exists) [14,38,39].

Fine needle aspiration cytological analysis has been proposed as an alternative to standard biopsy in the evaluation of azoospermic and severely oligozoospermic men [40]. This procedure has the advantage of being easily performed without anaesthesia on both testes, the analysis can be done in few hours, and the prognostic value for subsequent sperm retrieval by testicular sperm extraction (TESE) is very high. However, it is available only in few centres.

## 7. Indication for Additional Tests and Sperm Analyses

In cases of suspected retrograde ejaculation, post-ejaculation urine analysis should be performed [2,3,5]. Complete retrograde ejaculation causes aspermia (no semen) and cannot be easily distinguished from the absence of ejaculation, whereas low semen volume can be the result of partial retrograde ejaculation. Retrograde ejaculation is relatively infrequent among general infertile males, but it is particularly frequent in diabetic patients (due to autonomic neuropathy), men with spinal cord injury, patients who had undergone transurethral resection of prostate or open prostatectomy, and some neurological conditions.

A number of tests have been proposed to assess sperm quality and function, as well as DNA integrity, such as protamination and DNA packaging, DNA fragmentation, chromosome aneuploidy, mitochondrial function, apoptosis, and telomere length [22,41,42,43]. These tests, mainly sperm DNA fragmentation, although not approved for routine investigation, could give additional information in specific conditions, such as recurrent abortion or repeated ART failure, especially when gross abnormalities on standard semen analysis are not present [3,44]. Some authors suggested also that a large (grade 2–3) varicocele in the setting of normal semen parameters, or a small (grade 1) varicocele in the setting of equivocal semen parameters could be a possible indication for sperm DNA analysis [45]. However, a number of problems still limit their routine use in the investigation of the infertile man [22,44]. In fact, the usefulness of these methods in the evaluation of male factor infertility and as prognostic markers for natural fertility and pregnancy after ART is still debatable, although some studies are in favor that DNA damage helps in determining the male factor infertility in a couple. Controversies exist, including problems related to the different methods proposed and their standardization. Furthermore, normal values are yet under investigation, and prospective randomized controlled studies have not been performed. For the limitations outlined above, current guidelines do not mention many of these tests or they do not support their routine application [3,46], and we agree that sperm DNA fragmentation could be useful to date only in infertile couple with recurrent pregnancy loss from natural conception and ART and idiopathic infertile men with complete normozoospermia. Furthermore, sperm DNA fragmentation could be useful to select patients who might benefit from nutraceutical/antioxidant therapy [15,47], as it represents a marker of oxidative stress.

## 8. Conclusions

Etiological and pathophysiological diagnosis of male factor infertility is often complicated and sometimes inconclusive despite all the efforts put in the diagnostic workup, as described in this article and summarized in Table 7. The interaction with the female partner, the number of possible causes and risk factors (and many of them with limited evidence as disruptors of male fertility potential), and our still inadequate knowledge on the physiology and pathophysiology of spermatogenesis and sperm function make the diagnosis of male infertility a challenging path. The modern andrologist should have expertise in many fields, from genetics to microbiology, to urology or psychology, and should work in strict contact with other professionals to apply at best the medicine of reproduction.

On the other hand, the great success of assisted reproduction and related laboratory techniques erroneously promoted the concept that it is sufficient to have few sperm for ART to say that the andrologist made his work. In this way, sadly, it is too frequent that a diagnosis of male factor infertility is not performed at all, and investigation of the male partner is limited to some parameters of semen analysis [48]. As discussed in this article, the diagnosis of male infertility is not equivalent to semen analysis, and rational treatment is not ART. Infertility should always be viewed as a possible symptom of a more general or constitutional disease [12], and men should be investigated accordingly.

It is quite obvious that a complete, accurate and in-depth diagnostic process allowing the characterization of the etiological and pathophysiological diagnosis of the male factor directs treatments for the couple infertility. The definition of a clear diagnosis for the male partner guides prognosis and treatment, which should consider lifestyle, removing causative factors, rational medical or surgical treatments, and empirical treatments in the other cases, in a personalized way related to the particular couple and considering the health and wellness of offspring conceived [1,14,15].

## Figures and Tables

**Table 1:** Risk factors for infertility that should prompt for a full examination irrespective of the duration of infertility. Major risk factors are those for which the evidence and literature is strong in supporting a role for male infertility and they have a profound effect. Minor risk factors are those for which evidence is weaker or the impact on male infertility is not obligate.

Major Risk Factors	Minor Risk Factors
Cryptorchidism Testicular hypotrophy Testicular cancer Known genetic factors (e.g., karyotype anomalies, cystic fibrosis, thalassemia) Varicocele Testicular trauma Reproductive tract infections Testicular torsion Iatrogenic causes (pelvic and inguinal surgery, chemotherapy, radiotherapy, medications) Systemic diseases and/or endocrine diseases (e.g., diabetes mellitus, renal diseases, hepatic disease) Anabolic steroid use Pubertal disorders Infertility with previous partners	Environmental and/or occupational exposition Aging Cigarette smoking Alcohol and substances of abuse Obesity Genital heat stress Repeated abortion Testicular microlithiasis Family history for infertility and repeated abortion

**Table 2:** Scheme of history taking for the male partner of an infertile couple.

**General data**	Age
	Religion
	Primary or secondary infertility
	Duration of infertility
	Fertility history with previous partners
**Family history**	Infertility
	Repeated abortion
	Fetal malformation
	Genetic disorders
**Past medical history**	Known genetic factors
	Cancers and related treatments
	Medications
	Systemic diseases
	Endocrine diseases
	Anabolic steroid use
	Pubertal disorders
**Reproductive and urinary tract diseases**	Cryptorchidism
	Testicular cancer
	Varicocele
	Testicular trauma
	Reproduction tract infections (orchitis, epididymitis, prostato-vesiculitis)
	Testicular torsion
**Surgery**	Pelvic and abdominal surgery (prostate, bladder)
	Inguinal surgery (orchidopexy, orchiectomy, inguinal hernia)
	Varicocelectomy
	Hydrocele
	Vasectomy
	Vasovasostomy, vasoepididymostomy
**Occupational and lifestyle history**	Exposure to possible endocrine disruptors, heavy metals,
	electromagnetic fields
	Cigarette smoking
	Alcohol and substances of abuse
	Diet (high-energy diet, fat, and fried food consumption)
	Genital heat stress (tight fitting underwear, sauna use)
**Sexual history**	Timing and frequency of sexual intercourses
	Libido
	Erections
	Ejaculation

**Table 3:** Scheme of physical examination for an infertile male.

**General physical examination**	Height, weight, body mass index, waist circumference
	Muscle and fat distribution
**Genital examination**	Penis (overall anatomy, curvature, plaque, urethral meatus, condylomas, glans inflammation)
	Testes (location, volume by Prader orchidometer, consistency, nodules, hydrocele)
	Epididymes and vas deferens (presence, calibre, cysts, pain at palpation)
	Palpable varicocele, Valsalva manoeuver
**Secondary sex characteristics**	Gynecomastia
	Distribution of pubic hair
	General hair growth and distribution
	Body proportion
**Digital rectal examination**	Prostate (volume, nodules, pain)

**Table 4:** Lower reference limits (5th percentiles and their 95% confidence intervals) for semen parameters [16].

Parameter	Lower Reference Limit
Semen volume (mL)	1.5 (1.4–1.7)
Total sperm number (million per ejaculate)	39 (33–46)
Sperm concentration (million per mL)	15 (12–16)
Total motility (progressive + non-progressive, %)	40 (38–42)
Progressive motility (%)	32 (31–34)
Vitality	58 (55–63)
Sperm morphology (normal forms, %)	4 (3.0–4.0)
**Other reference values**	
pH	≥7.2
Peroxidase-positive leukocytes (million per mL)	<1.0

**Table 5:** Nomenclature related to semen analysis (modified from reference [16]).

**Normozoospermia**	Total number (or concentration, depending on outcome reported) * of spermatozoa, and percentages of progressively motile (PR) and morphologically normal spermatozoa, equal to or above the lower reference limits
**Quantitative alterations**	
Azoospermia	No spermatozoa in the ejaculate (after pellet analysis after centrifugation)
Cryptozoospermia	Spermatozoa absent from fresh preparations but observed in a centrifuged pellet
Oligozoospermia	Total number (or concentration, depending on outcome reported) * of spermatozoa below the lower reference limit
**Qualitative sperm alterations**	
Asthenozoospermia	Percentage of progressively motile (PR) spermatozoa below the lower reference limit
Necrozoospermia	Low percentage of live (and high percentage of immotile) spermatozoa in the ejaculate
Teratozoospermia	Percentage of morphologically normal spermatozoa below the lower reference limit
**Mixed alterations**	
Asthenoteratozoospermia	Progressively motile (PR) and morphologically normal spermatozoa below the lower reference limits
Oligoasthenozoospermia	Total number (or concentration) of spermatozoa and progressively motile (PR) spermatozoa below the lower reference limits
Oligoasthenoteratozoospermia	Total number (or concentration) of spermatozoa, progressively motile (PR) and morphologically normal spermatozoa below the lower reference limits
Oligoteratozoospermia	Total number (or concentration) of spermatozoa and morphologically normal spermatozoa below the lower reference limits
**Other**	
Haemospermia (haematospermia)	Presence of erythrocytes in the ejaculate
Leukospermia (leukocytospermia, pyospermia)	Presence of leukocytes in the ejaculate above the threshold value

* Preference should always be given to total number.

**Table 6:** Significance of hormonal levels in infertile men with quantitative and/or qualitative semen alterations. FSH: follicle stimulating hormone, LH: luteinizing hormone, MAGI: male accessory gland infection.

FSH	LH	Testosterone	Interpretation	Example of Aetiology
Normal	Normal	Normal	Post-testicular forms	Absence/obstruction of vas deferens; retrograde ejaculation
			Mild primary testicular forms: unilateral pathologies mild bilateral pathologies	Varicocele, orchiectomy Systemic diseases, lifestyle
			Qualitative sperm alterations	MAGI, antisperm antibodies
High	High	Low-normal	Primary testicular forms (spermatogenesis and Leydig cell damage)	Klinefelter syndrome, chemoradiotherapy
High	Normal	Normal	Primary testicular forms (only spermatogenesis is damaged)	Y chromosome microdeletions, cryptorchidism
Low	Low	Low	Pre-testicular (central, hypothalamic-pituitary) forms	Congenital and acquired hypogonadotropic hypogonadism
Low	Low	High	Pre-testicular (central, hypothalamic-pituitary) forms	Anabolic steroid use
High	High	High	Mixed forms	Androgen resistance (androgen receptor mutations)
Low	Normal	Normal	Low FSH	FSHβ gene mutations

**Table 7:** Summary of investigation of the male partner of an infertile couple or men who question their fertility status.

Step and Goal	Methods	Interpretation
Risk factor assessment (Table 1, Table 2 and Table 3)	History and physical examination	If risk factors are present, second and/or third-line investigation should be added to initial evaluation.
Fertility potential assessment, initial investigation (Table 4 and Table 5)	Fertility status of the partner	Evaluation of the couple fertility potential is essential to direct the diagnostic process of the male.
	Semen analysis	Other than information on fertility potential, it gives evidence on the status of the entire seminal tract, therefore giving indications for further analyses other than fertility assessment.
		Normozoospermia is not synonymous of normal fertility, as well as alterations in semen analysis are not synonymous of infertility.
		If abnormalities in semen analysis are found (quantitative or qualitative sperm defects or semen fluid alterations) indications for diagnosis, pathophysiology, and treatment could be derived only considering them together with further analyses.
		No therapies should be initiated based on semen analysis alone.
Full investigation for definition of aetiology, pathophysiology and to have prognostic and therapeutic information (Table 6 and Table 7)	Semen microbiological examination Endocrine assessment Imaging	Analyses should be performed in a personalized way, based on risk factor assessment, semen analysis, and evaluation of the fertility potential of the partner.
		Semen microbiological examination, endocrine assessment and imaging are useful in most cases of infertile men.
		At minimum, semen analysis, testicular volumes, and endocrine assessment should guide on the origin of the problem (pre-testicular, testicular, post-testicular).
	Testicular histology/cytology	Testicular histology/cytology should be done especially in cases of azoospermia and severe oligozoospermia.
		Exclusively diagnostic testicular histology is not recommended: testicular sperm cryopreservation should be done at the same time.
	Genetic testing	Routine genetic testing includes karyotype and Y chromosome microdeletions in cases of non-obstructive azoospermia and severe oligozoospermia, and *CFTR* mutation analysis in cases of bilateral and unilateral absence of vas deferens.
		Other specific genetic tests should be done based on specific clinical indications, and include *AR*, *NR5A1*, *TEX11*, dynein genes *DNAI1*, *DNAH5*, *DNAH11*, *DPY19L2*, *AURKC*, *INSL3*, *RXFP2*, and genes for hypogonadotropic hypogonadism.
		Polymorphism in *FSHB* and *FSHR* might have pharmacogenetic value for FSH treatment, but are not approves for routine use.
Additional tests (not yet approved for routine use) to have information in cases of unexplained infertility	Sperm DNA fragmentation	These tests, although not approved for routine investigation, could give additional information in specific conditions, such as recurrent abortion or repeated ART failure, especially when gross abnormalities on standard semen analysis are not present.
	Sperm aneuploidies and molecular karyotype	
	Sperm functional tests (protamination, mitochondrial function, apoptosis)	
	Strict criteriafor morphological evaluation of sperm	
